# Sheathless Size-Based Acoustic Particle Separation

**DOI:** 10.3390/s120100905

**Published:** 2012-01-16

**Authors:** Rasim Guldiken, Myeong Chan Jo, Nathan D. Gallant, Utkan Demirci, Jiang Zhe

**Affiliations:** 1 Department of Mechanical Engineering, University of South Florida, Tampa, FL 33620, USA; E-Mails: mjo@mail.usf.edu (M.C.J.); ngallant@usf.edu (N.D.G.); 2 Center for Biomedical Engineering, Department of Medicine, Brigham and Women’s Hospital, Harvard Medical School, Boston, MA 02115, USA; E-Mail: utkan@mit.edu; 3 Harvard-MIT Health Sciences and Technology, Cambridge, MA 02139, USA; 4 Department of Mechanical Engineering, University of Akron, Akron, OH 44325, USA

**Keywords:** standing surface acoustic wave, sheathless, size-based, separation

## Abstract

Particle separation is of great interest in many biological and biomedical applications. Flow-based methods have been used to sort particles and cells. However, the main challenge with flow based particle separation systems is the need for a sheath flow for successful operation. Existence of the sheath liquid dilutes the analyte, necessitates precise flow control between sample and sheath flow, requires a complicated design to create sheath flow and separation efficiency depends on the sheath liquid composition. In this paper, we present a microfluidic platform for sheathless particle separation using standing surface acoustic waves. In this platform, particles are first lined up at the center of the channel without introducing any external sheath flow. The particles are then entered into the second stage where particles are driven towards the off-center pressure nodes for size based separation. The larger particles are exposed to more lateral displacement in the channel due to the acoustic force differences. Consequently, different-size particles are separated into multiple collection outlets. The prominent feature of the present microfluidic platform is that the device does not require the use of the sheath flow for positioning and aligning of particles. Instead, the sheathless flow focusing and separation are integrated within a single microfluidic device and accomplished simultaneously. In this paper, we demonstrated two different particle size-resolution separations; (1) 3 μm and 10 μm and (2) 3 μm and 5 μm. Also, the effects of the input power, the flow rate, and particle concentration on the separation efficiency were investigated. These technologies have potential to impact broadly various areas including the essential microfluidic components for lab-on-a-chip system and integrated biological and biomedical applications.

## 1. Introduction

The separation of particles or cells is critical for many biological and biomedical applications, including cell biology, diagnostics and therapeutics. For instance, efficient separation and quantification of human T-lymphocytes (CD4+) from whole blood is necessary for the monitoring and treatment of HIV [1]. Also, the diagnostic test for malaria depends on the separation of parasite infected red blood cells from uninfected cells [2]. Particle separation is particularly important for multichannel resistive/inductive pulse microparticle sensors [3–5] whose sensitivity is proportional to the ratio of particle/microchannel size. For these types of sensors, size-dependent particle separation would allow different-sized micro/nano particles to be guided to different sensing channels with corresponding sizes, thus greatly improving the sensitivity and dynamic range at a high throughput. To date various techniques for particle or cell separation have been studied including hydrodynamic filtration [6,7], deterministic lateral displacement [8,9], pinched flow fractionation [10,11], sedimentation [12], inertial [13,14], magnetophoresis [15,16], negative magnetophoresis [17,18], optical [19,20] and dielectrophoresis [21,22].

Separation of biological particles, e.g., cells, can be performed using acoustic forces. When particles in a medium are subjected to an acoustic wave field, they experience pressure gradients. These pressure gradients can be used to manipulate suspended particles [23–26]. The acoustic-based method is an ideal particle or cell manipulation method for lab-on-a-chip devices, since this label-free method features low manufacturing cost, non-invasive nature, ability to separate a vast number of particles, and fast response time. Recent studies demonstrated the separation and manipulation of particles in microchannels using bulk acoustic waves generated by substrate-bonded bulk transducers [27–29]. However, the generation of bulk acoustic waves requires a high acoustic reflection coefficient of the microfluidic channel material. This requirement makes the bulk acoustic wave approach not applicable to many microfluidic devices that utilize commonly used materials with poor acoustic reflection such as polydimethylsiloxane (PDMS) [30]. Furthermore, since bulky commercial transducers are employed to generate acoustic waves, it conflicts with miniaturization and integration efforts.

To alleviate these shortcomings, surface acoustic wave (SAW) based techniques have been investigated owing to their low propagation loss, high sensitivity to the surface modification, low power consumption, and facile integration into microfluidic networks. One concern about the acoustic based separation technology is that the mechanical forces generated by the acoustic waves may potentially damage cells. However, since the operating frequencies of SAW separation devices (tens of MHz) correspond to time scales smaller than the molecular relaxation time and certainly any relaxation time of the cellular structures, there is no shear damage with these high frequency devices [31,32]. SAW devices have been investigated in wide variety of applications including fluid-mixing [33,34], fluid-pumping [35,36], particle focusing [30,37], and particle sorting/collection [38,39] in microchannels. Recently, standing surface acoustic waves (SSAW), generated by interdigitated microelectrodes on a piezoelectric substrate, have been demonstrated to separate polystyrene microparticles [40]. The technique of using SSAWs that travel along the substrate surface works with any microchannel material and can be incorporated easily into a multi-functional device design due to its simplicity.

However, current state-of-the-art SSAW based particle separation platform employs two external sheath flows to divide particle mixture streams as well as to prevent trapping and aggregating along the sidewall of the channel [40]. Introducing sheath flow to a microchannel has several fundamental disadvantages, such as dilution of the analyte by the sheath liquid, need for accurate flow control between sample and sheath flow, complicated structure in order to create sheath flow, and separation efficiency strongly depending on the sheath liquid composition [41]. Previous studies have investigated sheathless 3D particle focusing [37] and pattering acting as acoustic tweezers [42] using SSAW without any particle/cell separation capability. Most recently, Nam *et al.* [43] has demonstrated a size-dependent particle separation using SSAW. However, the device still uses two sheath flows to align particles toward the central region of the microfluidic channel. As a result, currently there is no report of SSAW based particle separation technique without using any external sheath flow. In this paper, we present a sheathless SSAW based particle separation technique in a microfluidic channel. Using a novel two-stage design, the microparticles are continuously separated at the outlet without using an external sheath flow.

## 2. Working Principle

**Figure 1.**
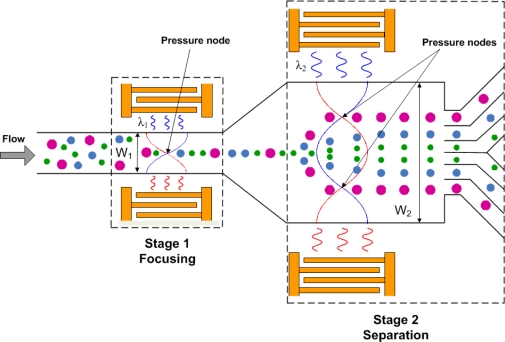
Conceptual view of the sheathless particle separator using standing surface acoustic waves. The first stage aligns the particles on the center line, while the second stage separates them according to size.

Figure 1 shows the design concept and working mechanism of the two-stage SSAW particle separator. The first stage uses a relatively narrow channel to align particles at the center of the microfluidic channel without introducing any external sheath flow. Two identical interdigitated transducers (IDTs) are fabricated on a piezoelectric substrate, and a microfluidic channel is aligned between the IDTs. When the two IDTs are stimulated with RF signals of equal amplitude, two series of surface acoustic waves propagate in opposite directions toward the particle solution inside the microchannel. The interference of the two surface acoustic waves forms a SSAW that generates a periodic distribution of pressure nodes and anti-nodes inside the microchannel. When the surface acoustic waves reach the liquid medium inside the microchannel, they are converted to leakage waves resulting in pressure fluctuations in the medium. The acoustic radiation forces caused by the pressure fluctuations move the particles toward the pressure nodes in the SSAW field. In the first stage, the width of the microchannel (W_1_) is chosen to be the half-wavelength (λ_1_/2) of the SSAW so that the channel contains only one pressure node located in the center of the channel (Figure 1). Thus, particles will aggregate at the center-line of the microchannel by the time they reach the end of the first stage microchannel. In the second stage, a wider channel (W_2_) that has a width of one-wavelength of the SSAW (λ_2_), so that two off-center pressure nodes exist in the channel. Suspended particles enter the second stage channel at the anti-node. Thus the acoustic forces will move particles towards the pressure nodes. Because the acoustic force on a particle is proportional to its volume, large particles will move to the pressure nodes more quickly than small particles during a given short SSAW exposure time. Therefore the particles can be separated by size.

A particle in an SSAW field is subjected to an acoustic radiation force, which can be expressed as [44]:

(1) $$F_{r}=-\frac{\left(\pi p_{o}{}^{2}V\beta_{m}\right)}{2\lambda }\phi (\beta ,\;\rho )\text{sin}\;(2kx)$$

(2) $$\phi (\beta ,\;\rho )=\frac{\left(5\rho_{p}-2\rho_{m}\right)}{\left(2\rho_{p}+\rho_{m}\right)}-\frac{\beta_{p}}{\beta_{m}}$$

where, *p*_o_, *V*, λ, *β*, *ρ*, *k*, *x* are the acoustic pressure amplitude, particle volume, wavelength, compressibility, density, wave number, and the distance from the pressure node respectively. The subscripts of *p* and *m* denote particle and liquid medium, respectively. Acoustic contrast factor (∅) determines whether the particle will move towards the pressure node or the anti-node: if ∅ > 0, particles will be attracted to the pressure node; if ∅ < 0, particles will aggregate at the anti-node. In general, most solid particles including cells in liquid medium move towards pressure node [45].

**Figure 2.**
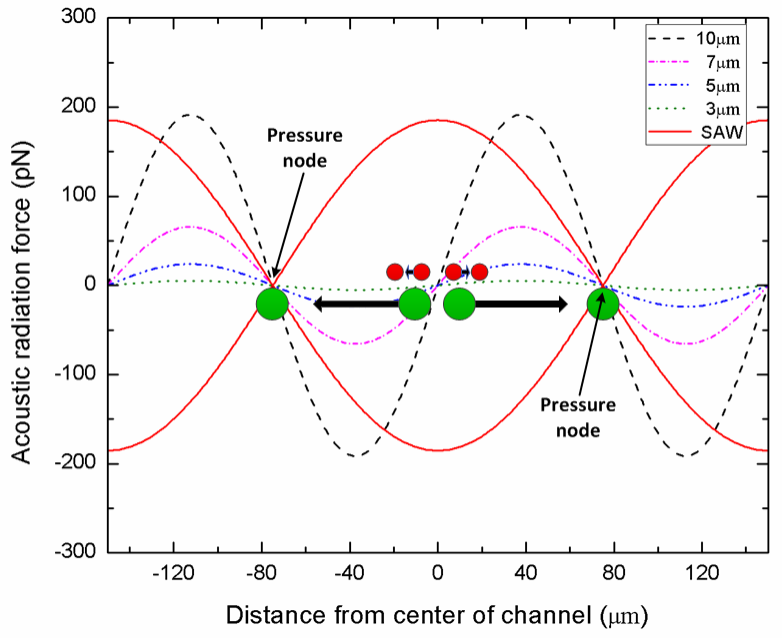
The distribution of acoustic radiation forces within the 2nd stage channel.

Figure 2 shows the acoustic radiation force distribution as a function of particle size in the microchannel of the second stage. The acoustic radiation forces were represented by Equations (1) and (2) under the presented experiment conditions (ρ = 1.05 g/cm^3^ and β = 2.46e−10 Pa^−1^ for polystyrene particles, ρ = 1.0 g/cm^3^ and β = 4.58e−10 Pa^−1^ for DI-water medium, wavelength = 300 μm, input power = 0.5 W). The results show that the acoustic forces change sinusoidally and are equal to zero at the wave crest, wave trough and nodal plane. Especially, as the particle diameter is reduced, the acoustic force decreases very rapidly. The peak value of the acoustic force of 10 μm particle is 191 pN, while that of 3 μm particle is only 5 pN. Since the particle displacements induced by acoustic forces are strongly dependent on the particle size, the larger particles are moved to the pressure nodes, whereas smaller particles remain in the center stream at a given SAW working time. It is important to note that our approach is capable of particle separation based on not only size, but also by density, compressibility, or acoustic contrast factor.

**Figure 3.**
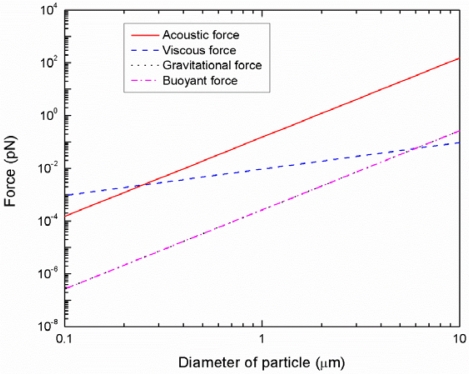
Theoretical analysis of forces acting on particles as a function of particle size.

The particles or cells suspended in a medium are subjected to four different forces: acoustic radiation force, viscous drag force, gravity force, and buoyant force. Figure 3 illustrates theoretically calculated magnitude of these forces as a function of particle size at the present experimental parameters (ρ = 1.05 g/cm^3^ and β = 2.46e−10 Pa^−1^ for polystyrene particles, ρ = 1.0 g/cm^3^ and β = 4.58e−10 Pa^−1^ for DI-water medium, wavelength = 300 μm, input power = 0.5 W). It can be observed that the gravitational and buoyant forces are balanced with similar magnitudes and opposite directions. Since the viscous force is proportional to the radius of the particles (*r*) while the acoustic force is proportional to the volume (*r*^3^) of the particles, the acoustic forces are generally dominant in the case of larger particles. However, when the diameter of the particles is less than about 0.3 μm, the acoustic forces are smaller than the viscous forces. In this case, the size-dependant separation may not be achieved by acoustic radiation forces.

**Figure 4.**
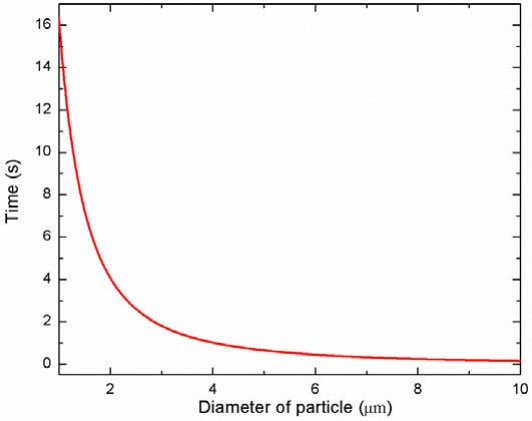
Time required for the particle migration toward the pressure node as a function of particle size.

The time required for the particle migration toward the pressure node can be theoretically predicted by a quantitative force analysis [40,42]. Figure 4 shows the time required for the particle migration toward the pressure node with varying the diameters of the particles. As expected, the larger particle moves to the pressure node faster than the smaller particle (for instance, 3 μm: 1.81 s, 5 μm: 0.65 s, 10 μm: 0.16 s). Based on these predictions, the length of the microchannel in each stage can be determined. The channel length of the first stage should be long enough so that all particles can reach the pressure node at the center of the channel, while the channel length of the second stage should be relatively short so that only larger particle move to the pressure nodes. It is important to note that the actual migration time of the particles toward the pressure node can also be readily adjusted by tuning the input power and flow rate during the experiments.

## 3. Device Design and Fabrication

As presented in Table 1, the SAW wavelength, the IDT finger pitch, and finger width were chosen as 300 μm, 300 μm, and 75 μm, respectively. The channel width of the first stage was 150 μm, half of the SAW wavelength, to contain only one pressure node in the center of the channel. On the other hand, the second stage width was 300 μm, the same as SAW wavelength, to have two pressure nodes in the channel. The resonance frequency of the SAW is determined by the ratio of the SAW velocity (*V_SAW_*) on the substrate and SAW wavelength (λ); *f = V_SAW_/λ*. With the SAW velocity 3,990 m/s for the chosen SAW direction on the substrate, the resonance frequency is 13.2 MHz.

Nam *et al.* have also reported that the pressure nodes would appear in the two-dimensional plane along the axial flow in a microchannel as the direction of SSAW is perpendicular to the axial flow direction [43]. Such two-dimensional particle distribution would be dependent on the channel height or aspect ratio of channel. For a channel with low aspect ratio, the flow velocity is practically uniform over the channel width except near the edges, while having a parabolic profile over the shorter dimension [46]. Thus, thin rectangular channel would be more efficient for applying SSAWs on particles. As a result, the microchannel height is chosen as 100 μm ensuring a low aspect ratio (1st stage: H/W = 0.66, 2nd stage: H/W = 0.33) for increased separation efficiency.

**Figure 5.**
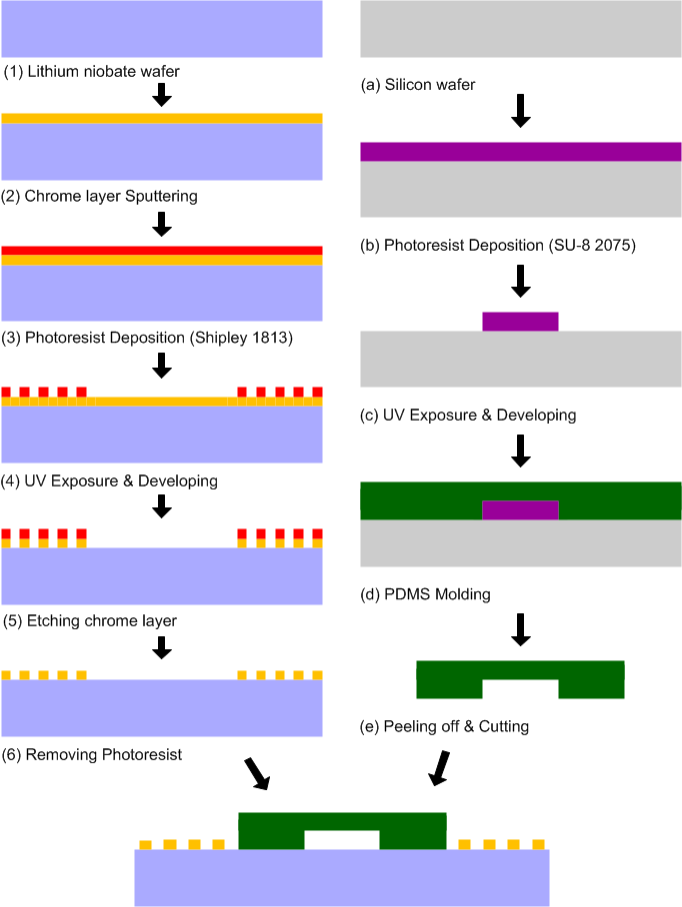
Fabrication process flow of sheathless acoustic particle separator using SSAW.

**Figure 6.**
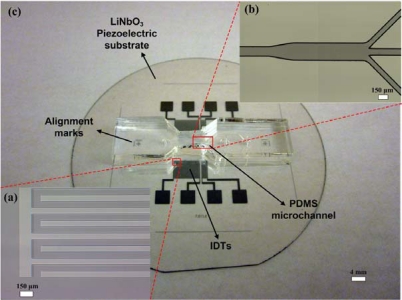
(**a**) Fabricated IDTs patterned on a lithium niobate wafer; (**b**) Fabricated SU-8 microchannel mold; (**c**) Completed sheathless acoustic particle separator.

The sheathless acoustic particle separator was realized by three consecutive fabrication steps: the fabrication of IDTs on a substrate, the fabrication of the PDMS microchannel, and the bonding of the PDMS microchannel to the substrate containing IDTs. Figure 5 shows the fabrication process flow of the present sheathless acoustic particle separator. For the fabrication of the IDT substrate, a two-side polished Y + 128° X-propagation lithium niobate (LiNbO_3_) wafer was used, as it has a high electromechanical coupling coefficient [47]. The wafer was first pre-cleaned by rinsing with acetone, methanol and de-ionized water. A 100 nm thick chrome layer was then deposited on the lithium niobate wafer using CRC sputtering system (Torr International, New Windsor, NY, USA). The lithium niobate wafer was then spun with 1.6 μm-thick photoresist (S1813, Shipley, Marlborough, MA, USA) at 3,000 rpm for 40 s, and soft baking of the photoresist was performed on a hot plate at 100 °C for 1 min. The wafer was patterned with a UV light source with an exposure dose of 125 mJ/cm^2^ and developed in a photoresist developer (MF 319, Shipley) for 70 s. After hard baking was performed on a hot plate at 115 °C for 5 min, the chrome layer was etched using chrome etchant (CR-7S, Cyantek, Fremont, CA, USA). Finally, the photoresist was removed by the photoresist remover. Each IDT consisted of 20 finger pairs with 150 μm finger pitch and 75 μm finger width. The sizes of IDT regions fabricated on the first stage and the second stage are 7.7 mm × 6 mm and 1.7 mm × 6 mm, respectively. The fabricated IDTs on the lithium niobate substrate are shown in Figure 6(a).

The microchannel was fabricated with a PDMS micromolding technique. To obtain 100 μm thick mold layer, negative photoresist (SU-8 2075, MicroChem, Newton, MA, USA) was spun onto the silicon wafer at 500 rpm for 10 s with acceleration of 100 rpm/s to spread out the photoresist, then at 2,000 rpm for 30 s with acceleration of 300 rpm/s. The wafer was then soft baked for 5 min at 65 °C and 20 min at 95 °C. The wafer was then patterned with a UV light source with an exposure dose of 240 mJ/cm^2^ and post exposure baking was performed directly after exposure for 5 min at 65 °C and 10 min at 95 °C. After developed in the SU-8 developer for 10 min, the wafer was hard baked for 5 min at 150 °C. The uniformity of the SU-8 mold height was confirmed using a surface profilometer. The PDMS oligomer and crosslinking prepolymer of the PDMS agent from a Sylgard™ 184 kit (Dow Corning, Midland, MI, USA) was mixed in a weight ratio of 10:1, poured onto the SU-8 mold, and then cured at room temperature for 24 h to prevent PDMS shrinking due to heat. After the PDMS replica was peeled off from the mold, the inlets and outlets were generated using 20-gauge needle. The fabricated microchannel mold is shown in Figure 6(b). For bonding the PDMS microchannel to the substrate containing IDTs, oxygen plasma (1 min at 20 sccm oxygen flow rate, 500 mTorr chamber pressure, and 100 W power) was used to activate both surfaces. After ethanol, acting as a lubricant, was dropped on the surface of IDT substrate, alignment of the PDMS microchannel and IDT substrate was conducted. Figure 6(c) shows a complete sheathless acoustic particle separator including the PDMS microchannel and IDT substrate.

## 4. Experimental Setup

**Figure 7.**
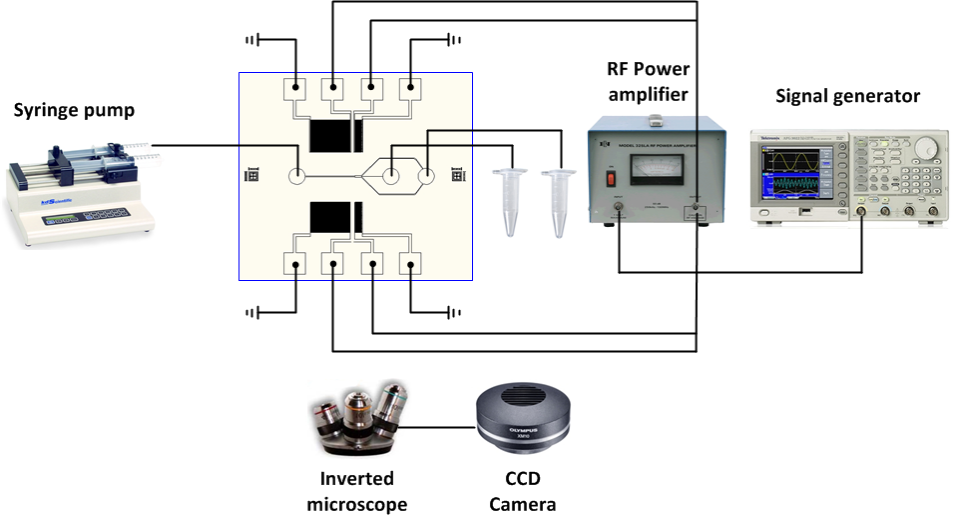
Experimental setup for the sheathless acoustic particle separation.

An experiment was conducted with the device on an inverted microscope (IX-51, Olympus). A mixture solution of polystyrene fluorescent particles (Thermo Scientific, Waltham, MA, USA) with diameters of 3 μm and 10 μm diameter (ρ = 1.05 g/cm^3^ and β = 2.46e−10 Pa^−1^ for polystyrene particles, ρ = 1.0 g/cm^3^ and β = 4.58e−10 Pa^−1^ for DI-water medium) was injected into the microchannel by a syringe pump (KDS200, KD Scientific, Holliston, MA, USA). Also, for high-resolution particle separation experiments, a mixture solution of polystyrene fluorescent particles with diameters of 3 μm (red) and 5 μm (green) diameters was used. An AC signal was generated by a signal generator (AFG3022B, Tektronix) and then amplified by an RF power amplifier (325LA, ENI). The signal was split two ways to provide identical signals to the IDTs and generate SSAW. The high speed images of fluorescent in the microchannel were obtained with a CCD camera (XM-10, Olympus) and image acquisition software (cellSens, Olympus). A complete experimental setup is shown in Figure 7.

To study the effect various parameters on the separation efficiency, the input power range applied to the IDTs was from 250 mW to 1 W, the flow rate ranged from 0.5 μL/min to 5 μL/min, and the concentration of each of the particles in the sample suspension ranged from 1% to 4% by volume. The PDMS microchannel was responsible for the range of the power applied in the present experiments. The net power applied to the IDTs to achieve particle focusing and separation depends on the SAW wavelength, particle size, and flow speed. Prior studies of SAW induced by IDT have applied similar power values to PDMS microchannel for particle manipulation [23,32,43].

## 5. Results and Discussion

**Figure 8.**
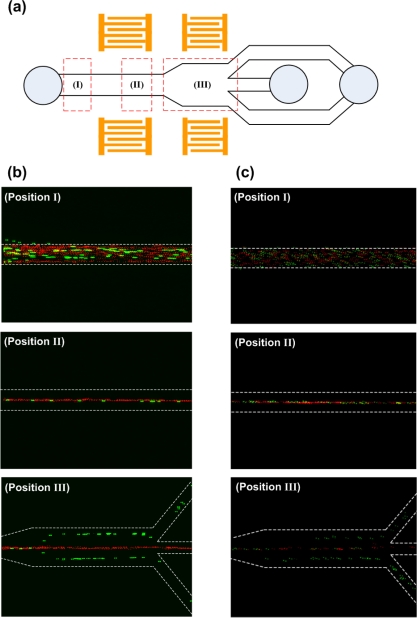
(**a**) The chosen location (I–III) in the test section for recording the fluorescent images of the each particle stream; (**b**) Fluorescent images of 10 μm (green) and 3 μm (red) particles distribution. Constant operating frequency of 13.2 MHz, input power of 1 W, flow rate of 0.5 μL/min, and particle concentration of 1% by volume were applied; (**c**) Fluorescent images of 5 μm (green) and 3 μm (red) particles distribution. Constant operating frequency of 13.2 MHz, input power of 1.45 W, flow rate of 0.2 μL/min, and particle concentration of 1% by volume were applied.

At three different locations marked as (I), (II) and (III) in Figure 8(a), the distribution of 3 μm and 10 μm PS particle streams was captured during the separation process as shown in Figure 8(b). During the experiment, the frequency, the input power, the flow rate, and the concentration were set to 13.2 MHz, 1 W, 0.5 μL/min, and 1% by volume, respectively. Location (I) was before the particles entered the first stage. One can clearly observe that both sizes of particles were randomly scattered in the channel. When particles entered the first stage (location (II)), the acoustic forces acting on the particles aligned them at the center of the channel where pressure nodes existed. Location (III) coincided with the second stage of the device. As can be observed from the figure of the location (III), the 10 μm particles moved to the pressure nodes while the 3 μm particles remained in the center of the channel because the acoustic forces exerted on the 3 μm particles were insufficient to push them into the pressure nodes during a given short SSAW exposure time. As a result, the larger particles were separated to the side channels and the smaller particles were directed to the center channel.

Figure 8(c) shows the distribution of 3 μm and 5 μm polystyrene particle streams during the separation process at the same locations as illustrated in Figure 8(a). Since the acoustic forces were not applied to the inlet region, expectedly both particle sizes were randomly distributed in the channel (location (I)). As the particles entered the first stage, they were accumulated and aligned by the acoustic forces to the center of the channel where the pressure node existed (location (II)). When the particles traveled to the second stage (location (III)), 5 μm particles did not migrate completely to the pressure nodes located near side walls and remained in the center stream due to insufficient acoustic forces during short SSAW exposure time. On the other hand, when the input power was significantly increased even 3 μm particles are driven to the pressure nodes, conflicting with our separation efforts. As a result, an optimization study has been conducted to determine the appropriate input power and the flow rate parameters to accomplish high-resolution separation with small size difference particle streams. As illustrated in Figure 8(c) location III, with the optimized input power parameter of 1.45 W and flow rate of 0.2 μL/min, 5 μm particles were separated to the side channels while 3 μm particles were collected to the center channel. These experiments show that the present device is capable of separating different-size particles without introducing any external sheath flow to the microchannel.

**Figure 9.**
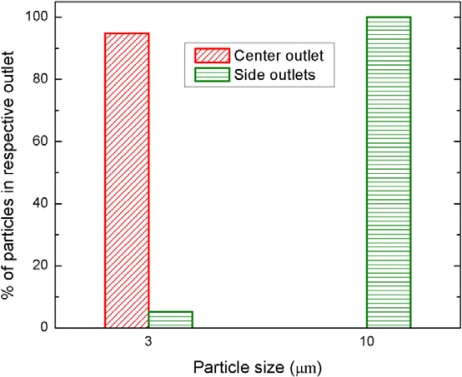
Distribution of each of the two particle sizes (3 and 10 μm) over the two outlets.

To quantitatively determine the separation efficiency, the images were acquired from each outlet at 20 frames per second using CCD camera. The acquired images (600 frames) were analyzed with image processing software (ImageJ^®^, National Institutes of Health, Bethesda, MD, USA) to count the number of particles collected from each outlet. First, the images were converted to grayscale and then the threshold was set for each image. The thresholded images were then converted to binary images. Since some of particles were agglomerated, automatic thresholding method recognized them as a single object. Thus, a watershed segmentation process was performed to accurately count such cases. Finally, the number of particles was quantified using the menu command *Analyze particles* with the specific parameters. Figure 9 shows the percentage of each of the two size particles collected from each outlet. The graph demonstrates that all 10 μm particles (100%) were separated to the side outlets, while most 3 μm particles (94.8%) remained in the center channel.

**Figure 10.**
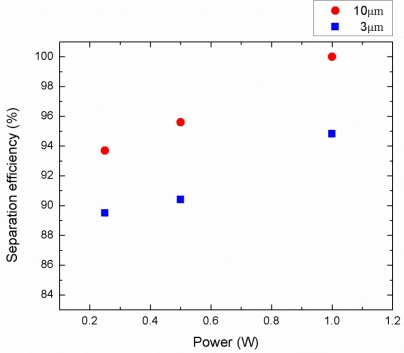
Separation efficiency as a function of input power for 3 and 10 μm particles (driving frequency: 13.2 MHz, flow rate: 0.5 μL/min, particle concentration: 1% by volume).

**Figure 11.**
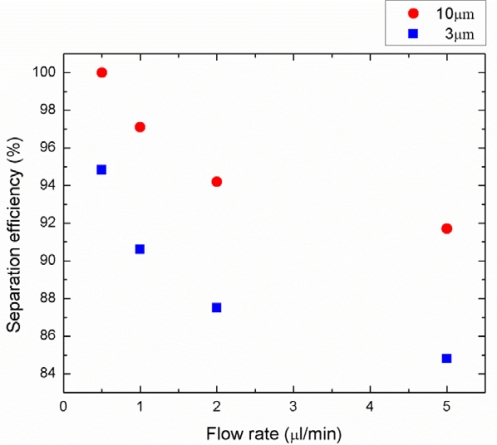
Separation efficiency as a function of flow rate for 3 and 10 μm particles (driving frequency: 13.2 MHz, input power: 1 W, particle concentration: 1% by volume).

**Figure 12.**
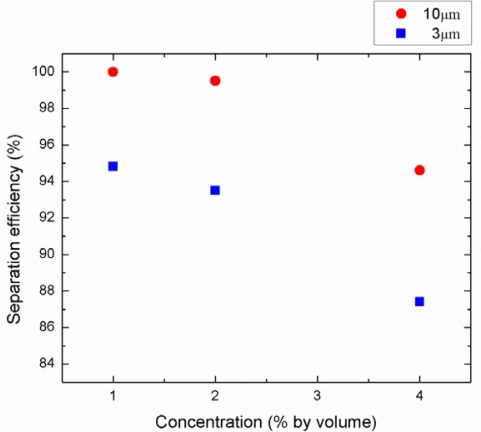
Separation efficiency as a function of particle concentration for 3 and 10 μm particles (driving frequency: 13.2 MHz, input power: 1 W, flow rate: 0.5 μL/min).

The acoustic separation efficiency depends on the applied power, flow rate, wavelength of the SAW, channel geometry and particle concentration. Among these parameters, we investigated the effects of the input power, the flow rate, and particle concentration on the separation efficiency as illustrated in Figures 10, 11, and 12 respectively. The separation efficiency was defined as A/(A + B) for the 3 μm particles and B/(A + B) for the 10 μm particles, where A is the number of the target particle collected from the center outlet and B is the number of the target particle collected from the side outlets.

As a result of the particle counting analysis, the separation efficiency for both particle sizes as a function of power applied to IDTs is shown in Figure 10. The driving frequency was 13.2 MHz, the particle concentration was 1% by volume, and the flow rate was fixed at 0.5 μL/min. As can be observed from this figure, the separation efficiency of the 3 μm and the 10 μm particles were measured in the range of 89.5–94.8% and 93.7–100%, respectively, at the applied power range of 0.25 to 1 W. The separation efficiency increased as the input power increased because the acoustic radiation force is proportional to the acoustic pressure amplitude which is determined by the input power and acoustic impedance. The effect of the flow rate on the particle separation efficiency is also investigated in Figure 11. The driving frequency, particle concentration, and the applied power were kept constant at 13.2 MHz, 1% by volume, and 1 W, respectively. The separation efficiencies of the 3 μm and the 10 μm particles were obtained in the range of 84.6–94.8% and 91.7–100%, respectively, at the flow rate range of 0.5 to 5 μL/min. The results show that lower flow rates provide higher separation efficiency since the particles were exposed to the SSAW field for a longer time period allowing higher number of particles moving to the pressure nodes. Experimental efficiency results with different particle concentrations were also shown in Figure 12. Constant driving frequency of 13.2 MHz, input power of 1 W, and a flow rate of 0.5 μL/min were used during these experiments. The separation efficiencies of the 3 μm and the 10 μm particles were achieved in the range of 87.4–94.8% and 94.6–100%, respectively, at the particle concentration range of 1 to 4%. The higher particle concentration yielded lower separation efficiency because the width of the aligned particles band at the first stage increased and existence of the thicker 3 μm particle band slowed down the displacement of the 10 μm particles across the microchannel in the second stage. As a result, the separation of very high concentration bioparticles such as whole blood with this platform may be more involved and may require additional power input or lower flow rate with the wider channel outlets to achieve high separation efficiency.

As expected, the separation efficiency of the 3 μm particles was lower than that of the 10 μm particles for all cases. The fundamental reason for this behavior was that the number of the misaligned 3 μm particles at the first stage was more than that of the 10 μm particles due to the relatively low acoustic forces exerted on the 3 μm particles. In the separation procedure of this platform, the alignment of the particle is one of the most important tasks directly affecting the efficiency of the separation. If the particles don’t line up at the center of the channel before entering the second stage, this can negatively impact the separation efficiency, as illustrated by our own experiments. It is worth commenting here that this particular design is only capable of separating two different size particle streams. However, as shown in Figure 2, the peak values of the acoustic force on 3 μm, 5 μm, 7 μm, and 10 μm particles are 5 pN, 24 pN, 66 pN, and 191 pN, respectively. This force magnitude difference is sufficient to achieve three or more different size particle separation. Hence by modifying the working wavelength, the IDT configuration, the width of the channel, and the number of the channel outlets separation of three or more different size particle streams can be achieved with this platform.

It is important to note that this particle separator is ideal for the microparticle sensor because (1) it is applicable to all types of solid particles regardless of their size, shape, and electrical/magnetic/optical properties; (2) it is a non-invasive method and requires low power intensity; (3) it allows the use of a relatively large microfluidic channel in comparison to particle separation using pinched flow fractionation [6,11,48] and deterministic lateral displacement [49–51], which will allow for high throughput and cause less problems with clogging, and most importantly; (4) it does not require the use of sheath flow, which usually change the composition and concentration of the analyte. Our future efforts will be aimed at using this platform for separating biological objects such as cells.

## 6. Conclusions

In summary, sheathless SSAW based particle separation using a novel two-stage microfluidic platform has been demonstrated. Two polystyrene fluorescent particles with different diameters (3 and 10 μm) were successfully separated with high efficiency. The prominent feature of the present microfluidic platform is that the device does not require the use of the sheath flow for positioning and aligning of particles in order to separate particles. The sheathless flow focusing and separation are integrated within a single microfluidic device and accomplished simultaneously. This device sets the first demonstration of SSAW based separation in a microfluidic channel to eliminate sheath flows. We anticipate that this sheathless acoustic particle separation method will find broad applications in wide variety of biological and biomedical applications.

## Figures and Tables

**Table 1.** Design parameters used for the size-based separation experiments.

Wavelength (λ)	300 μm	First channel width	150 μm
IDT finger width	75 μm	Second channel width	300 μm
IDT finger pitch	150 μm	Channel height	100 μm
IDT finger pairs	20 pairs	Resonance frequency	13.2 MHz

## References

[1] Moon S., Gurkan U.A., Blander J., Fawzi W.W., Aboud S., Mugusi F., Kuritzkes D.R., Demirci U. (2011). Enumeration of CD_4_(^+^) T-cells using a portable microchip count platform in tanzanian HIV-infected patients. PLoS One.

[2] Gascoyne P., Satayavivad J., Ruchirawat M. (2004). Microfluidic approaches to malaria detection. Acta Trop.

[3] Jagtiani A.V., Carletta J., Zhe J. (2011). A microfluidic multichannel resistive pulse sensor using frequency division multiplexing for high throughput counting of micro particles. J. Micromech. Microeng.

[4] Zhe J., Jagtiani A., Dutta P., Hu J., Carletta J. (2007). A micromachined high throughput coulter counter for bioparticle detection and counting. J. Micromech. Microeng.

[5] Du L., Zhe J.A., Carletta J., Veillette R., Choy F. (2010). Real-time monitoring of wear debris in lubrication oil using a microfluidic inductive Coulter counting device. Microfluid. Nanofluid.

[6] Yamada M., Seki M. (2005). Hydrodynamic filtration for on-chip particle concentration and classification utilizing microfluidics. Lab Chip.

[7] Yamada M., Kano K., Tsuda Y., Kobayashi J., Yamato M., Seki M., Okano T. (2007). Microfluidic devices for size-dependent separation of liver cells. Biomed. Microdevices.

[8] Huang L.R., Cox E.C., Austin R.H., Sturm J.C. (2004). Continuous particle separation through deterministic lateral displacement. Science.

[9] Long B.R., Heller M., Beech J.P., Linke H., Bruus H., Tegenfeldt J.O. (2008). Multidirectional sorting modes in deterministic lateral displacement devices. Phys. Rev. E.

[10] Larsen A.V., Poulsen L., Birgens H., Dufva M., Kristensen A. (2008). Pinched flow fractionation devices for detection of single nucleotide polymorphisms. Lab Chip.

[11] Yamada M., Nakashima M., Seki M. (2004). Pinched flow fractionation: Continuous size separation of particles utilizing a laminar flow profile in a pinched microchannel. Anal. Chem.

[12] Huh D., Bahng J.H., Ling Y.B., Wei H.H., Kripfgans O.D., Fowlkes J.B., Grotberg J.B., Takayama S. (2007). Gravity-driven microfluidic particle sorting device with hydrodynamic separation amplification. Anal. Chem.

[13] Di Carlo D. (2009). Inertial microfluidics. Lab Chip.

[14] Kuntaegowdanahalli S.S., Bhagat A.A.S., Kumar G., Papautsky I. (2009). Inertial microfluidics for continuous particle separation in spiral microchannels. Lab Chip.

[15] Jung J., Han K.H. (2008). Lateral-driven continuous magnetophoretic separation of blood cells. Appl. Phys. Lett.

[16] Han K.H., Frazier A.B. (2006). Paramagnetic capture mode magnetophoretic microseparator for high efficiency blood cell separations. Lab Chip.

[17] Zhu T.T., Marrero F., Mao L.D. (2010). Continuous separation of non-magnetic particles inside ferrofluids. Microfluid. Nanofluid.

[18] Kose A.R., Koser H. (2012). Ferrofluid mediated nanocytometry. Lab Chip.

[19] Shah G.J., Ohta A.T., Chiou E.P.Y., Wu M.C., Kim C.J. (2009). EWOD-driven droplet microfluidic device integrated with optoelectronic tweezers as an automated platform for cellular isolation and analysis. Lab Chip.

[20] MacDonald M.P., Spalding G.C., Dholakia K. (2003). Microfluidic sorting in an optical lattice. Nature.

[21] Chen D.F., Du H., Li W.H. (2006). A 3D paired microelectrode array for accumulation and separation of microparticles. J. Micromech. Microeng.

[22] Gascoyne P.R.C., Vykoukal J. (2002). Particle separation by dielectrophoresis. Electrophoresis.

[23] Tan M.K., Tjeung R., Ervin H., Yeo L.Y., Friend J. (2009). Double aperture focusing transducer for controlling microparticle motions in trapezoidal microchannels with surface acoustic waves. Appl. Phys. Lett.

[24] Kapishnikov S., Kantsler V., Steinberg V. (2006). Continuous particle size separation and size sorting using ultrasound in a microchannel. J. Stat. Mech.

[25] Nilsson A., Petersson F., Jonsson H., Laurell T. (2004). Acoustic control of suspended particles in micro fluidic chips. Lab Chip.

[26] Wiklund M., Gunther C., Lemor R., Jager M., Fuhr G., Hertz H.M. (2006). Ultrasonic standing wave manipulation technology integrated into a dielectrophoretic chip. Lab Chip.

[27] Demirci U., Montesano G. (2007). Single cell epitaxy by acoustic picoliter droplets. Lab Chip.

[28] Friend J., Yeo L.Y. (2011). Microscale acoustofluidics: Microfluidics driven via acoustics and ultrasonics. Rev. Mod. Phys.

[29] Jonsson H., Holm C., Nilsson A., Petersson F., Johnsson P., Laurell T. (2004). Particle separation using ultrasound can radically reduce embolic load to brain after cardiac surgery. Ann. Thorac. Surg.

[30] Li P.C.H., Wang W.J., Parameswaran M. (2003). An acoustic wave sensor incorporated with a microfluidic chip for analyzing muscle cell contraction. Analyst.

[31] Peterson F., Aberg L., Sward-Nilsson A.M., Laurell T. (2007). Free flow acoustophoresis: Microfluidic-based mode of particle and cell separation. Anal. Chem.

[32] Shi J.J., Mao X.L., Ahmed D., Colletti A., Huang T.J. (2008). Focusing microparticles in a microfluidic channel with standing surface acoustic waves (SSAW). Lab Chip.

[33] Tan M.K., Yeo L.Y., Friend J.R. (2009). Rapid fluid flow and mixing induced in microchannels using surface acoustic waves. Europhys. Lett.

[34] Tseng W.K., Lin J.L., Sung W.C., Chen S.H., Lee G.B. (2006). Active micro-mixers using surface acoustic waves on Y-cut 128 degrees LiNbO_3_. J. Micromech. Microeng.

[35] Cecchini M., Girardo S., Pisignano D., Cingolani R., Beltram F. (2008). Acoustic-counterflow microfluidics by surface acoustic waves. Appl. Phys. Lett.

[36] Girardo S., Cecchini M., Beltram F., Cingolani R., Pisignano D. (2008). Polydimethylsiloxane-LiNbO_3_ surface acoustic wave micropump devices for fluid control into microchannels. Lab Chip.

[37] Shi J.J., Yazdi S., Lin S.C.S., Ding X.Y., Chiang I.K., Sharp K., Huang T.J. (2011). Three-dimensional continuous particle focusing in a microfluidic channel via standing surface acoustic waves (SSAW). Lab Chip.

[38] Franke T., Braunmuller S., Schmid L., Wixforth A., Weitz D.A. (2010). Surface acoustic wave actuated cell sorting (SAWACS). Lab Chip.

[39] Tan M.K., Yeo L.Y., Friend J.R. (2010). Unique flow transitions and particle collection switching phenomena in a microchannel induced by surface acoustic waves. Appl. Phys. Lett.

[40] Shi J.J., Huang H., Stratton Z., Huang Y.P., Huang T.J. (2009). Continuous particle separation in a microfluidic channel via standing surface acoustic waves (SSAW). Lab Chip.

[41] Strege M.A., Lagu A.L. (2004). Capillary Electrophoresis of Proteins and Peptides.

[42] Shi J.J., Ahmed D., Mao X., Lin S.C.S., Lawit A., Huang T.J. (2009). Acoustic tweezers: Patterning cells and microparticles using standing surface acoustic waves (SSAW). Lab Chip.

[43] Nam J., Lee Y., Shin S. (2011). Size-dependent microparticles separation through standing surface acoustic waves. Microfluid. Nanofluid.

[44] Yosioka K., Kawasima Y. (1955). Acoustic radiation pressure on a compressible sphere. Acustica.

[45] Laurell T., Petersson F., Nilsson A. (2007). Chip integrated strategies for acoustic separation and manipulation of cells and particles. Chem. Soc. Rev.

[46] Stiles T., Fallon R., Vestad T., Oakey J., Marr D.W.M., Squier J., Jimenez R. (2005). Hydrodynamic focusing for vacuum-pumped microfluidics. Microfluid. Nanofluid.

[47] Gardner J.W., Varadan V.K., Awadelkarim O.O. (2001). Microsensors, MEMS, and Smart Devices.

[48] Yang S., Undar A., Zahn J.D. (2006). A microfluidic device for continuous, real time blood plasma separation. Lab Chip.

[49] Morton K.J., Loutherback K., Inglis D.W., Tsui O.K., Sturm J.C., Chou S.Y., Austin R.H. (2008). Hydrodynamic metamaterials: Microfabricated arrays to steer, refract, and focus streams of biomaterials. Proc. Natl. Acad. Sci. USA.

[50] Inglis D.W., Davis J.A., Austin R.H., Sturm J.C. (2006). Critical particle size for fractionation by deterministic lateral displacement. Lab Chip.

[51] Inglis D.W. (2009). Efficient microfluidic particle separation arrays. Appl. Phys. Lett.

